# Effects of a Mutation in the *HSPE1* Gene Encoding the Mitochondrial Co-chaperonin HSP10 and Its Potential Association with a Neurological and Developmental Disorder

**DOI:** 10.3389/fmolb.2016.00065

**Published:** 2016-10-07

**Authors:** Anne S. Bie, Paula Fernandez-Guerra, Rune I. D. Birkler, Shahar Nisemblat, Dita Pelnena, Xinping Lu, Joshua L. Deignan, Hane Lee, Naghmeh Dorrani, Thomas J. Corydon, Johan Palmfeldt, Liga Bivina, Abdussalam Azem, Kristin Herman, Peter Bross

**Affiliations:** ^1^Research Unit for Molecular Medicine, Aarhus University and Aarhus University Hospital Aarhus, Denmark; ^2^Department of Biochemistry & Molecular Biology, Tel Aviv University Tel Aviv, Israel; ^3^Department of Pathology and Laboratory Medicine, David Geffen School of Medicine at University of California, Los Angeles Los Angeles, CA, USA; ^4^Department of Pediatrics, David Geffen School of Medicine at University of California, Los Angeles Los Angeles, CA, USA; ^5^Department of Biomedicine, Aarhus University Aarhus, Denmark; ^6^Division of Genomic Medicine, Department of Pediatrics, UC Davis Health System Sacramento, CA, USA

**Keywords:** protein folding, molecular chaperones, mitochondrial proteins, neurological disorders, *De novo* mutations, oxidative stress

## Abstract

We here report molecular investigations of a missense mutation in the *HSPE1* gene encoding the HSP10 subunit of the HSP60/ HSP10 chaperonin complex that assists protein folding in the mitochondrial matrix. The mutation was identified in an infant who came to clinical attention due to infantile spasms at 3 months of age. Clinical exome sequencing revealed heterozygosity for a *HSPE1* NM_002157.2:c.217C>T *de novo* mutation causing replacement of leucine with phenylalanine at position 73 of the HSP10 protein. This variation has never been observed in public exome sequencing databases or the literature. To evaluate whether the mutation may be disease-associated we investigated its effects by *in vitro* and *ex vivo* studies. Our *in vitro* studies indicated that the purified mutant protein was functional, yet its thermal stability, spontaneous refolding propensity, and resistance to proteolytic treatment were profoundly impaired. Mass spectrometric analysis of patient fibroblasts revealed barely detectable levels of HSP10-p.Leu73Phe protein resulting in an almost 2-fold decrease of the ratio of HSP10 to HSP60 subunits. Amounts of the mitochondrial superoxide dismutase SOD2, a protein whose folding is known to strongly depend on the HSP60/HSP10 complex, were decreased to approximately 20% in patient fibroblasts in spite of unchanged SOD2 transcript levels. As a likely consequence, mitochondrial superoxide levels were increased about 2-fold. Although, we cannot exclude other causative or contributing factors, our experimental data support the notion that the HSP10-p.Leu73Phe mutation could be the cause or a strong contributing factor for the disorder in the described patient.

## Introduction

Heat shock protein 10 (HSP10) and heat shock protein 60 (HSP60) are the constituents of the HSP60/HSP10 chaperonin complex that assists folding of proteins in the mitochondrial matrix space (Cheng et al., 1989; Hartman et al., 1992). Chaperonins including the most thoroughly investigated *Escherichia coli* GroEL/GroES complex constitute a subfamily of the molecular chaperones characterized by the extraordinary architecture of these complexes that provides an inner cavity for folding of proteins (Horwich and Fenton, 2009; Hayer-Hartl et al., 2016). The HSP60 subunits are organized in a double-barrel structure that is built by two heptameric rings of 60 kDa subunits stacked back-to-back (Nisemblat et al., 2015). The inner cavity of the barrel initially binds unfolded proteins to the inner wall. Subsequent binding of a heptamer of HSP10 subunits puts a “lid” on the cavity. Binding of ATP molecules to the HSP60 subunits results in conformational changes of the HSP60/HSP10 complex (Saibil et al., 2013). Finally, timed ATP hydrolysis triggering additional conformational changes of the complex leads to dissociation of the HSP10 lid and discharge of the enclosed protein. One or several rounds of this process facilitate folding of interacting proteins. *In vitro* refolding studies have shown that under non-permissive conditions (i.e., spontaneous folding is minimal) the presence of HSP10 is strictly essential for efficient folding of model substrate proteins (Schmidt et al., 1994).

Together with other chaperones and proteases, the HSP60/ HSP10 complex forms the protein quality control (PQC) system in the mitochondrial matrix and its expression is regulated by the mitochondrial unfolded protein response and the heat-shock responses (Aldridge et al., 2007). PQC systems, also known as proteostasis networks (Balch et al., 2008), consist of molecular chaperones and proteases that collectively maintain the functional proteome by, on one hand assisting protein folding and on the other hand removing misfolded proteins, thus promoting folding to the native state and minimizing deleterious effects of misfolded proteins. PQC systems play a decisive role in many diseases including protein aggregation diseases like Alzheimer's and Parkinson's disease as well as protein misfolding diseases like phenylketonuria and medium-chain acyl-CoA dehydrogenase deficiency (Gregersen et al., 2006; Chen et al., 2011).

The mitochondrial matrix proteome is estimated to consist of at least 500 proteins (Rhee et al., 2013) that maintain mitochondrial ATP production, the energy fuel of cells. Furthermore, a plethora of other synthetic and important catalytic and regulatory functions (Raimundo, 2014) takes place in the mitochondrial matrix, which is the most protein-dense compartment in the cell.

The human HSP10 protein is encoded by the *HSPE1* gene that is located in a head to head arrangement with the *HSPD1* gene encoding HSP60. Both genes are transcribed under control of a bidirectional promoter localized between the genes (Ryan et al., 1997). The head to head arrangement of the *HSPD1* and *HSPE1* genes appears to secure transcription of both chaperonin genes at a fixed ratio and it is conserved from *Caenorhabditis elegans* to humans. Organization of the *E. coli groEL* and *groES* genes in an operon under control of a common promoter likewise has the same purpose (Ryan et al., 1997).

The neurological disorders hereditary spastic paraplegia SPG13 (Hansen et al., 2002) and MitCHAP-60 disease (Magen et al., 2008) are caused by missense mutations in the *HSPD1* gene (reviewed in Bross and Fernandez-Guerra, 2016). Hereditary spastic paraplegia SPG13 (OMIM #605280) is a late onset, autosomal dominantly inherited disorder that primarily affects motor neurons with the longest axons in the spinal cord. In contrast, the autosomal recessively inherited MitCHAP-60 disease (OMIM #612233) is linked to a much more severe fatal neurodegenerative disorder of early onset causing death within the first two decades of life and associated with highly pronounced cerebral hypomyelination. Investigations of the purified mutant HSP60 proteins associated with both diseases have indicated that these HSP60 mutant proteins are apparently normally incorporated into HSP60 ring complexes, but display reduced ATP hydrolysis activity and impaired refolding activity (Bross et al., 2008; Parnas et al., 2009).

*In vivo* studies in mice have shown that knock-out of both alleles of the *Hspd1* gene is embryonally lethal (Christensen et al., 2010). However, mice heterozygous for the knock-out allele recapitulate features of hereditary spastic paraplegia with late onset motoneuron disorder (Magnoni et al., 2013). These mice display swollen mitochondria in spinal cord, deficient complex III activity in spinal cord and brain cortex as well as increased protein carbonylation in these tissues suggesting increased ROS production (Magnoni et al., 2014). Importantly, complex III deficiency was found to be associated with decreased levels of the complex III subunit UQCRC1 and increased ROS levels appear to be due to impaired folding and increased turnover of the matrix superoxide dismutase SOD2. This suggested that HSP60 haploinsufficiency caused impaired folding of certain mitochondrial proteins thus leading to a variety of impaired functions. The different phenotypes caused by the different *HSPD1* mutant alleles and inheritance modes suggests that different types and degrees of disturbances of this system cause distinct phenotypic manifestations. The study of these genetic diseases is thus a unique opportunity to enlighten these basic mechanisms in humans and for pinpointing modes how to target these mechanisms for the treatment of diseases.

In the present study we investigated a patient with a history of infantile spasms, hypotonia, developmental delay, a slightly enlarged liver, macrocephaly, and mild non-specific dysmorphic features. Clinical exome sequencing revealed heterozygosity for a *de novo* point mutation in the *HSPE1* gene triggering studies of possible effects of the mutation at protein level and at cellular level in patient fibroblasts. As potentially disease-causing mutations in the *HSPE1* gene so-far only have been observed in this single patient, we cannot fully exclude other genetic and/or environmental causes, However, our results show that the investigated mutation causes malfunction of the HSP60/HSP10 complex. Taken together with the knowledge on diseases caused by mutations in the gene encoding the HSP60 subunit we make the case for a mutation disease relationship.

## Materials and methods

### Clinical patient analysis

The patient, a male infant, came to clinical attention due to infantile spasms at 3 months of age. Subsequent clinical investigations revealed hypotonia, developmental delay, a slightly enlarged liver, macrocephaly, and some mild nonspecific dysmorphic features. For more detailed description of the clinical picture see *Extended clinical patient information* in Appendix. Metabolic analysis on plasma, urine and cerebrospinal fluid (CSF) showed an unusual urine amino acid profile that did not fit a pattern of known metabolic disorders. Data on compounds that deviated from the control range in at least one analysis are shown in Table 1. It was characteristic for the metabolite analyses that values varied between repeated analyses suggesting that environmental factors and conditions may influence the metabolic profiles. Plasma amino acids and urine organic acids were overall normal. Urine dicarboxylic acids were slightly increased in two out of three analyses, which could indicate a fatty acid oxidation disorder. However, a plasma acyl-carnitine profile that had been determined prior to the first biochemical lab tests at age 6 months, showed no abnormalities.

**Table 1:** **Selected metabolite data**.

	**Compound**	**[mmol/mmol creatine]**	**Control range**	
			**Low**	**High**
Urine organic acids	3OH-Buturic	25/4/0	0	10
	Glutaric	6/3/2	0	5
	Acetoacetic	11/3/2	0	2
	Suberic	12/7/7	0	7
	OH-Dicanedioic	7/5/5	0	2
	Methylmalonic	2/6/5	0	5
	Octanoic	0/9/7	0	2
	Pyrovic	7/18/23	0	22
Urine amino acids	Gamma-aminobutyrate	143/71/107	0	11.9
	Hydroxy-lysine	5018/2273/1937	1.1	11
	Alpha-aminoadipic	212/64/202	5.1	30.3
	Beta-alanine	510/115/473	0	33.6
	Taurine	897/319/647	617	428.7
	Glutamine	181/173/151	75.8	176.7
	Halfcysteine	74/111/227	15.4	160.6
	Lysine	33/23/101	21.4	96.2
	**Compound**	μ**mol/L**	**Control range**	
			**Low**	**High**
Cerebrospinal fluid	Pyridoxal 5-Phosphate	22	23	64
	Free sialic acid	28	2	22
	Total sialic acid	60	8	50

*Urine organic acids and urine amino acid concentrations were measured as mmol/mol creatinine. Values from 3 measurements are given separated by “/.” Organic acids and amino acids concentrations in cerebrospinal fluid were measured as μmol/L. Only compounds that differed from control samples in at least one measurement are shown*.

Interestingly, the pyridoxal 5′-phosphate concentration in CSF was just below the reference interval and defective pyridoxine metabolism has been shown to cause seizures (Mills et al., 2006; Plecko et al., 2007). Furthermore, gamma aminobutyrate was elevated in urine, which could indicate GABA transaminase deficiency. GABA transaminase is dependent on pyridoxal 5′-phosphate and hence also related to the seizure symptoms. Imaging of the patient's brain was carried out with and without contrast (not shown), but no significant interval changes were detected, nor were there any mass effect, infarct, hemorrhage, demyelination or hydrocephalus. Because of mild hepatosplenomegaly noted at one point, lysosomal enzyme screening was carried out but results were inconspicuous.

Chromosomal microarray analysis showed a *de novo* 0.146 Mb deletion at 17q23.3 and a 0.316 Mb gain at 17q25.1 that was maternally inherited. The deletion involved a single gene, TANC2, which is most expressed in the adult brain and has very low expression in the fetal brain. It has not been associated with a known disorder to date. A review of the genes involved in the duplication did not show any genes that were suspected to be associated with the clinical features of this patient.

### Cell culturing

The patient fibroblast line was derived from a skin biopsy. Informed consent from the parents was obtained at the time of the skin biopsy and documented in the patient's medical record. Information included that the primary research focus would be to study the effect of the variant on the function of *HSPE1* and the potential impact it would have on the patient's clinical phenotype. Written informed consent from the family was also obtained. The investigation was performed in accordance with regulations set by the Danish National Committee on Health Research Ethics. Patient skin fibroblasts and three anonymized age-matched control fibroblasts were cultured according to standard procedures. In brief, fibroblasts were maintained in Dulbecco's modified Eagles media (DMEM), supplemented with 10% (v/v) fetal bovine serum, 29 mg/mL of L-glutamine (Leo Pharmaceutical), and 1% penicillin/streptomycin (Leo Pharmaceutical) at 37°C and 5% CO_2_. Fibroblasts were negative for *Mycoplasma sp*. and tests were performed routinely (PromoKine, Heidelberg, Germany)

### DNA and RNA purification

High molecular weight genomic DNA was isolated from whole blood by QIAcube (QIAGEN) according to manufacturer's protocol at UCLA Molecular Diagnostics Laboratories. RNA was isolated from fibroblast pellets by Trizol (Life Technologies) according to manufacturer's description. Quality and amounts of the DNA and RNA preparations was measured by NanoDrop Spectrophotometer (Thermo Scientific), Qubit (ThermoFisher Scientific), and agarose gel electrophoresis.

### DNA sequencing

Clinical exome sequencing and data analysis was performed at the UCLA Clinical Genomics Center on the patient and both parents (i.e., Trio-CES) following the CLIA (Clinical Laboratory Improvement Amendments) and CAP (College of American Pathologists) validated protocols (Lee et al., 2014). Briefly, exome capture was performed using the Agilent SureSelect Human All Exon 50 Mb kit (Agilent technologies) and HiSeq2000 (Illumina) as 50 bp paired end run. In the patient, total 13,377,501,340 bases of sequence were generated and uniquely aligned to the human reference genome, generating a mean depth of coverage of 150x per base within the RefSeq protein coding bases of the human genome with 94% of the bases covered at greater than 9 reads. Parental samples were sequenced at similar depth of coverage (mother: 159X; father: 152X). The *HSPE1* de novo variant was confirmed by Sanger sequencing at the UCLA Orphan Disease Testing Center (ODTC) in the trio.

Sanger sequencing of fibroblast genomic DNA and PCR products from cDNA derived from fibroblast RNA was performed using Big Dye® Terminator v.1.1 Cycle Sequencing Kit (Life Technologies, USA) and analysis with the Genetic Analyzer 3500 Dx (Life Technologies, USA). Sequence data were evaluated with Gensearch software (PhenoSystems, Belgium).

### Selected reaction monitoring (SRM)

Fibroblasts were lysed in 100 mM ammonium bicarbonate and 1 M urea with ultrasonication (Branson Sonifier 250, Branson Ultrasonics Corp) at output control 3 and 30 % duty cycle for three cycles of five pulses, with 1 min on ice between each cycle. Lysates were centrifuged at 13,000 *g* for 30 min at 4°C. Protein concentration of the soluble fraction was measured by Bradford Protein assay (Bio-Rad) and 30 μg were used for SRM analysis. Relative quantification of peptides was carried out using a modified version of the SRM assay described in Fernández-Guerra et al. (2014). In brief all targeted proteins were monitored by detection of 2–5 tryptic peptides. Defined amounts of heavy labeled synthetic peptide analogs were spiked into the samples and used for relative quantification. The summed fragment ion peak areas for each peptide were normalized to the signal responses from the corresponding spiked heavy-labeled peptide standards. The means of the ratios for each peptide measured in control fibroblasts were set to 100% and the means for the patient fibroblast samples were expressed as the percentage of these. Samples from 3 independently grown control fibroblasts and 3 parallel cultures of patient fibroblasts were analyzed.

### cDNA analysis and PCR

cDNA was synthesized from 1 μg of RNA using the iScript™ cDNA Synthesis Kit (BioRad). Subsequent qRT-PCR analysis was performed using TaqMan gene expression assays for *HSPE1, HSPD1, SOD2*, and *ACADM* and analyzed on ABI StepOne plus (Applied Biosystems) essentially as described in Hansen et al. (2008). Relative transcript levels were calculated using the standard curve method.

For sequencing of the mutation site on genomic DNA, a fragment was amplified with primers situated in intron 2 and the 3′-UTR of exon 4 of the *HSPE1* gene, respectively. For sequencing of the mutation site in cDNA derived from patient or control fibroblasts isolated RNA was amplified using primers in exon 2 and the coding region of exon 4. For primer sequences see Supplementary Table S1. Correct size of PCR products was analyzed by agarose gel electrophoresis.

### *In vitro* synthesis and mitochondrial import assay

*In vitro* transcription/translation and subsequent import into mitochondria was performed as described in Bross et al. (2003) with minor modifications. Briefly, cDNA sequences for wild type *HSPE1* and *HSPE1*_ c.217C>T in pcDNA3.1 plasmids (Eurofins) were used to produce HSP10-p.wt and HSP10-p.Leu73Phe protein by *in vitro* transcription/translation in rabbit reticulocyte lysate systems (TNT T7 kit, Promega) in the presence of [^35^S]-methionine as recommended by the supplier. Mitochondria were isolated from fresh mouse liver. Import mixtures were incubated at 37°C and aliquots were removed immediately after addition of the labeled HSP10 and after 15, 30, and 60 min. Fractions of the soluble proteins from mitochondrial lysates were analyzed by SDS PAGE (Criterion TGX gel, Any kDa, Biorad). Gels were stained with Coomassie, dried, exposed to phosphor imaging screens overnight and radiolabeled proteins were visualized by phosphor imaging (Typhoon, GE Healthcare).

### Cloning, mutagenesis and purification of HSP10-Leu73Phe protein

Human HSP10 cDNA (GenBank accession no. P61604) was inserted into the pET22b(+) expression plasmid (Novagen) using NdeI and XhoI restriction sites. A stop codon was inserted at the end of the cDNA sequence to generate a construct that does not contain a C-terminus His-tag. The Leu73Phe mutation was inserted into the HSP10 gene by site-directed mutagenesis according to the protocol of Stratagene, using the primers mHSP10L73F_F and m HSP10L73F_R (for primer sequences see Supplementary Table S1).

HSP10 was expressed as described in Parnas et al. (2009) and purified as follows: the cell pellet was resuspended (1:10 w/v) in a buffer containing 20 mM Tris-HCl pH 7.7, 5 mM MgSO4, 1 mM DTT, 1500 units DNase. Cells were homogenized and passed through a microfluidizer. Immediately after lysis, PMSF (0.5 mM) and 1 μg/ml of each of the following protease inhibitors (Sigma) were added: Pepstatin, Chymostatin, Antipain, Leupeptin, and Aprotinin. Debris was removed by centrifugation for 30 min at 35,000 *g*. The supernatant was loaded onto a RESOURCE Q column (GH Healthcare) equilibrated with buffer A (20 mM Tris-HCl pH 7.7, 0.1 mM EDTA, and 1 mM DTT). Unbound proteins were collected from the column and dialyzed over night against buffer B (20 mM MES pH 6.6 and 0.1 mM EDTA). The protein was then loaded on a SOURCE-S column (GH Healthcare) equilibrated with buffer B. Bound proteins were eluted from the column with a linear gradient of 0–500 mM NaCl (in buffer B). HSP10-enriched fractions were collected and concentrated. The protein was then loaded on a Superdex 200 prep grade gel-filtration column (Pharmacia) equilibrated with buffer C (50 mM Tris-HCl pH 7.7 and 100 mM NaCl). Fractions containing heptameric HSP10 were concentrated to approximately 40 mg/ml and flash-frozen in liquid nitrogen for storage. All stages were carried out at 4°C. Analysis of tryptic peptides by a nano Liquid-Chromatography system (Ultimate 3000, Dionex) coupled to a mass spectrometer (Q Exactive, Thermo Fisher Scientific) through an EASY-Spray nano-electrospray ion source (Thermo Scientific) confirmed high purity.

### Malate dehydrogenase (MDH) refolding assay

Refolding of HCl-denatured MDH was carried out as previously described by Bonshtien et al. (2009).

### Thermal unfolding/refolding kinetics

Temperature-dependent denaturation/renaturation experiments were performed using a ChirascanTM CD (CircularDichroism) spectrometer as described by Vitlin Gruber et al. (2013).

### Limited proteolysis assay

Purified HSP10-p.wt or HSP-p.Leu73Phe protein at 0.1 mg/ml in 100 mM Tris-HCl (pH 7.8) was incubated at 37°C. Trypsin was added to a final concentration of 0.01 mg/ml (molar ratio HSP10:trypsin 10:1). Samples were taken immediately before and 2, 10, 25, 60, and 120 min after addition of trypsin. Samples were quenched by addition of SDS PAGE sample buffer and incubation at 95°C for 2 min. As control, series without addition of trypsin were performed. Samples were subjected to SDS PAGE and proteins stained with Coomassie.

For analysis of the content of HSP10 bands from SDS PAGE, Coomassie–stained bands were excised and peptides extracted as described (Edhager et al., 2014). Peptides were analyzed using a nano Liquid-Chromatography system (Ultimate 3000, Dionex) coupled to a mass spectrometer (Q Exactive, Thermo Fisher Scientific) through an EASY-Spray nano-electrospray ion source (Thermo Scientific).

### Image cytometry and bioenergetics measurements

All cytometric fluorescent measurements were performed using the NC-3000 image cytometer (Chemometec) as described in Fernandez-Guerra et al. (2016). Mitochondrial oxygen consumption rate profiling assays of cultured fibroblasts were performed using a Seahorse XF^e^96 extracellular flux analyzer and the Mito Stress test kit (Seahorse Bioscience) as recommended by the supplier with the following modifications: 15,000 cells per well were seeded 24 h before analysis and FCCP was added at 1 μM final concentration. Oxygen consumption rate (OCR) was normalized to total protein amount measured in each well after the analysis using the Bradford Protein assay (Bio-Rad).

### Mitochondrial morphology

The mitochondrial morphology of fibroblast from the patient and healthy individuals was analyzed using Mitotracker Green FM (Molecular Probes, Life Technologies). Fibroblasts from the patient and healthy individuals were seeded at 50% confluence in 6-well plates (Nunc, Roskilde, Denmark) 24 h before the analysis. The fibroblasts were stained with 100 nM Mitotracker Green (Molecular probes) for 30 min at 37°C. Pictures were obtained with the EVOS FLoid Cell Imaging Station (Life Technologies) with the standard green channel of the instrument (482/18 nm excitation and 532/59 nm emission). The image was adjusted for contrast with the software of the instrument; no further processing of the image was done.

## Results

### Clinical exome sequencing reveals a *de novo* mutation in the *HSPE1* gene encoding the HSP10 subunit of the mitochondrial HSP60/HSP10 chaperonin complex

As clinical and metabolic investigations of the patient did not reveal the causative factor of the symptoms (for information on the patient see Materials and Methods *Clinical patient analysis* and Appendix “*Extended clinical patient information”*), whole exome sequencing on the patient and the parents was performed as the next step. No relevant mutations were found in a list of 758 genes (Supplementary Table S2) annotated with keywords relevant for the disease phenotype. However, further analysis of the exome sequencing data revealed a *de novo* heterozygous missense variation, c.217C>T [p.Leu73Phe], in the *HSPE1* gene encoding the mitochondrial co-chaperonin HSP10 that forms part of the HSP60/ HSPSP10 chaperonin complex. Presence in the patient and absence in the parents was confirmed by Sanger sequencing. The detected variation has to our knowledge never before been reported in the literature or publicly accessible genetic databases and the *HSPE1* gene has not previously been associated with a human disorder. Variation in the third position of the CTC codon for the mutated leucine-73 of the *HSPE1* gene (to T or G) resulting in codons that also specify leucine has been observed as documented by the Exome Aggregation Consortium (ExAC; Cambridge, MA; accessed December 2015). Investigation of the HSP10 mutation *in silico* using the prediction tool PolyPhen2 predicted the mutation to be “possibly damaging,” with a score of 0.954 on a scale from 0 to 1. Similarly, the SIFT prediction tool (Ng and Henikoff, 2001) predicted the mutation to be “damaging.”

**Figure 1:**
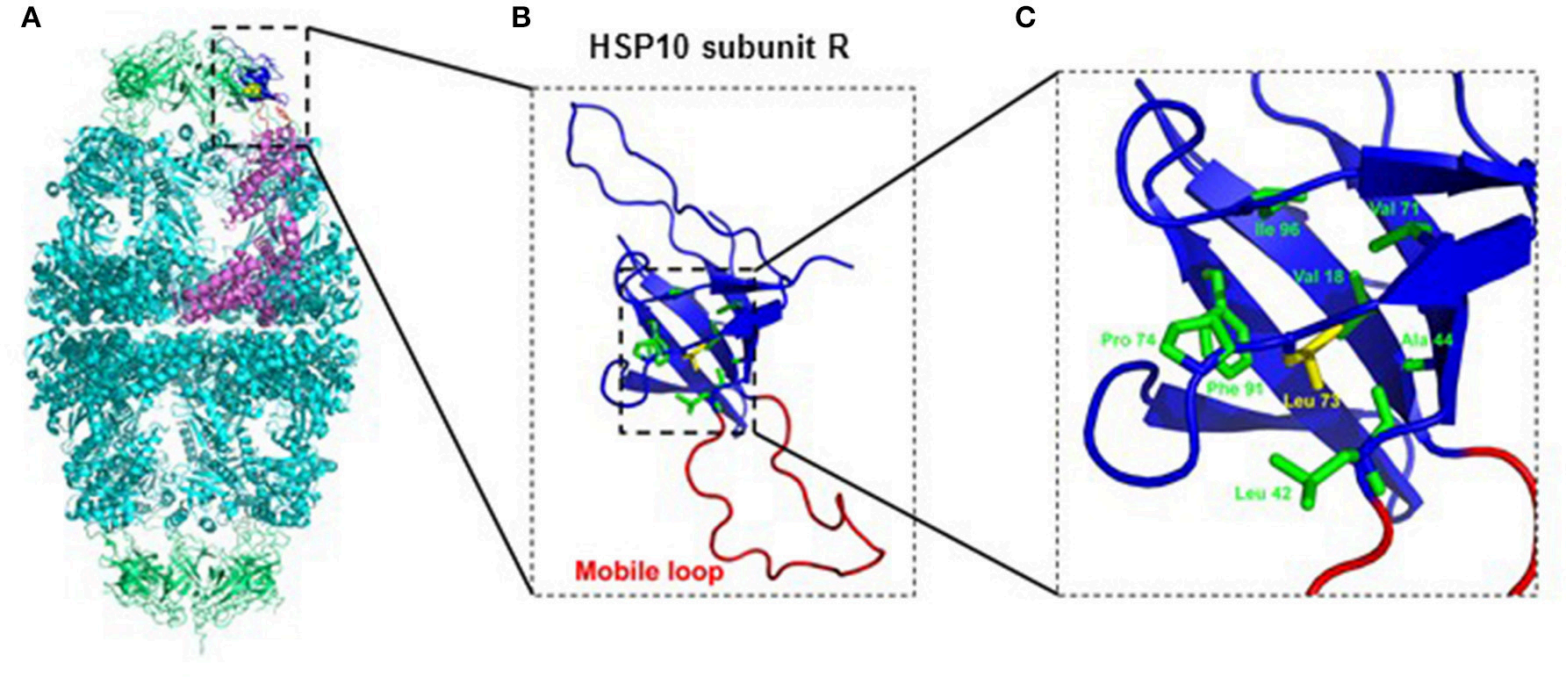
**Position of Leucine-73 in the HSP60/HSP10 complex structure. (A)** HSP60-HSP10 symmetric football complex shown in cartoon representation. HSP60 subunits are in cyan and HSP10 subunits are in green. One HSP60 subunit (subunit D) and its interacting HSP10 subunit (subunit R) are emphasized (purple and blue respectively). The mobile loop of subunit R is in red. Leucine-73 residue is depicted in yellow with space filling representation. **(B)** Enlarged view of the boxed area in **(A)** displaying HSP10's subunit R. Color scheme as in **(A)**. Leucine-73 and its interacting residues at the hydrophobic core of HSP10 (green) are presented as sticks. **(C)** Enlarged view of the boxed hydrophobic core of HSP10 from figure **(B)**. Leucine-73 and its interacting residues are labeled. Leucine-73 is facing the hydrophobic core made by the beta-sheets building HSP10 main body. In addition, analysis of Leucine-73 interactions in all HSP10 subunits using the PISA and PIC servers (Krissinel and Henrick, 2007; Tina et al., 2007) showed that Leucine-73 is tightly packed in the core of the HSP10 molecule. It has an average accessible surface area of only 1.36 Å^2^, while it interacts with the following residues of the core: Val18, Leu42, Ala44, Val71, Pro74, Phe91, and Ile96. Illustrations were generated using the PyMOL program (The PyMOL Molecular Graphics System, version 1.5.0.4; Schrödinger, LLC; available at www.pymol.org), with PDB ID: 4PJ1.

Inspection of the crystal structure of the HSP60/HSP10 complex (Figure 1) showed that the mutated leucine-73 is located distantly from the mobile loop region that mediates interaction with HSP60 and the mutation is thus not expected to directly affect this interaction. Leucine-73 is facing the hydrophobic core made by the beta-sheets building the HSP10 main body (Figure 1). When we examined leucine-73 solvent accessibility using the PISA program (Krissinel and Henrick, 2007) we noticed that the average accessible surface area (ASA) of this residue is only 1.36 Å^2^ indicating that it is tightly packed. The neighboring Leu72 for instance has an average ASA of 53.3 Å^2^, while Leu97 that is positioned between HSP10 subunits has an ASA of more than 120 Å^2^.

### *In vitro* studies of the mutation effect

**Figure 2:**
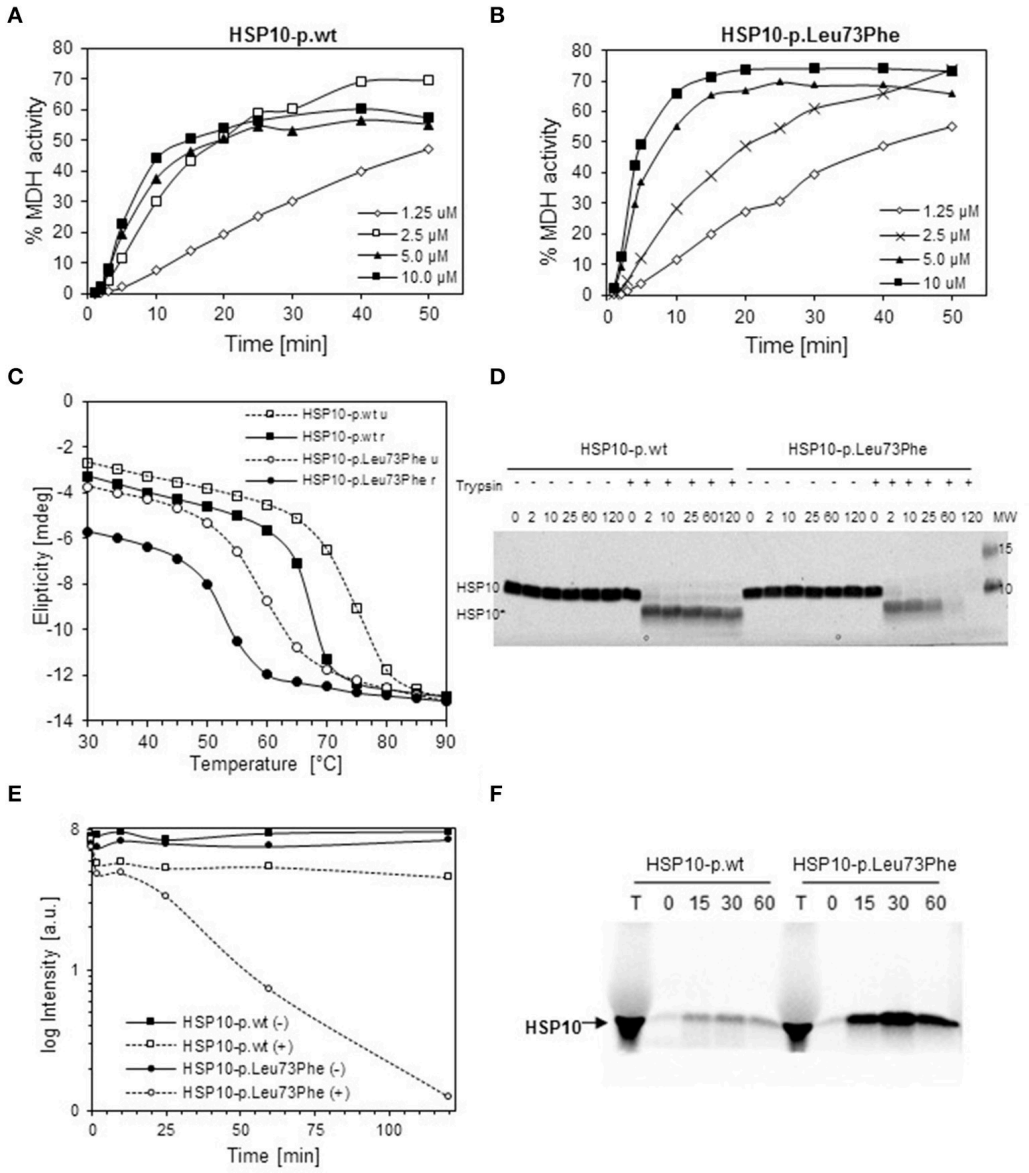
*****In vitro*** properties of recombinantly produced HSP10-p.Leu73Phe. (A,B)** Refolding kinetics of denatured MDH. HCl-denatured MDH (0.33 mM) was refolded at 30°C with HSP60 (10 μM), ATP (1 mMol/L) and the indicated concentrations of wild type **(A)** or p.Leu73Phe mutant **(B)** HSP10. Refolding is expressed as the percentage of MDH activity before denaturation. **(C)** Thermal unfolding/refolding kinetics of wild type and p.Leu73Phe mutant HSP10. Folding status was monitored by following the circular dichroism signal at 222 nm. 0.3 mg/ml HSP10-p.wt or HSP10-p.Leu73Phe in buffer containing 50 mM Tris-HCl pH 7.7 and 100 mM NaCl were analyzed. The experiments were carried out by varying the temperature from 25 to 90°C (stippled lines; u = unfolding) and back to 25°C (solid lines; r = refolding). Ellipticity was measured with 2°C intervals, 180 s setting time and 120 s time per point. **(D)** Limited proteolysis of HSP10 proteins with trypsin. HSP10-p.wt and HSP10-Leu73Phe at 0.1 μg/ml in 100 mM Tris-HCl (pH 7.8) were incubated at 37°C with or without addition of trypsin at a stoichiometric ratio of 1:10 (trypsin: HSP10). Proteolysis was stopped with addition of SDS PAGE sample buffer at the time points indicated and proteins were separated by SDS PAGE and stained with Coomassie. The positions of full-length HSP10 and HSP10 core fragment are indicated. **(E)** Quantification of the band-intensities from the Coomassie-stained gel shown in **(D)**. **(F)** Import kinetics of wild type and p.Leu73Phe mutant HSP10 into isolated mouse liver mitochondria. The HSP10 proteins were synthesized and radioactively labeled using a reticulocyte lysate system programmed by plasmids carrying the respective cDNAs as described in Materials and Methods. Translated proteins were added to freshly isolated mouse liver mitochondria and incubated at 37°C. Aliquots were taken at the time points indicated, and mitochondria were trypsin-treated and reisolated. Aliquots were run on SDS PAGE followed by phosphorimaging. T: aliquot from the *in vitro* translation reaction.

To explore the effects of the mutation on structural and functional properties of the co-chaperonin, we performed *in vitro* studies on purified HSP10-p.Leu73Phe protein following recombinant expression in bacteria. We analyzed the function of HSP10-p.Leu73Phe *in vitro* by testing its ability to assist HSP60 in refolding of malate dehydrogenase (MDH). MDH was denatured and diluted into a solution with HSP60 to allow its binding to the chaperonin. Then ATP and different concentrations of either HSP10-p.wt (Figure 2A) or HSP10-p.Leu73Phe (Figure 2B) were added to let the folding reaction proceed. Surprisingly, the results showed that the HSP10-p.Leu73Phe protein was fully active and even had a tendency to display slightly faster kinetics in assisting MDH refolding. However, when comparing the different HSP10 concentrations, we noticed that the reaction with HSP10-p.wt was already saturated at 2.5 μM HSP10 protein whereas HSP10-p.Leu73Phe had to be present at 5 μM to saturate the reaction (Figures 2A,B).

We then investigated the thermal stability of the HSP10-pLeu73Phe protein *in vitro*. Thermal unfolding and refolding of mutant and wild type HSP10 proteins was monitored by CD spectroscopy. Figure 2C shows a representative experiment. The HSP10-p.Leu73Phe protein unfolds with a Tm of only 59°C compared to HSP10-p.wt that displays a Tm of approximately 73°C (Figure 2C; stippled lines). A 14° difference between the Tm's of wild type and mutant HSP10 would result in a reduction of the free energy of unfolding of approximately 3 kcal (Greenfield, 2006). Considering that the free energy difference between the unfolded and folded conformations of proteins is in the range of 5–20 kcal (Pace, 1990), we conclude that the conformational stability of the HSP10 mutant protein is significantly decreased.

We also examined the reverse reaction of refolding wild type and mutant HSP10 by gradually decreasing the temperature from 90° to 25°C. Both proteins spontaneously refold upon decreasing the temperature (Figure 2C; solid lines). The Tm of refolding of both the wild type and mutant HSP10 has shifted to lower temperatures compared to their respective Tm for unfolding. Interestingly the HSP10-p.Leu73Phe protein regained a lower level of refolding at 25°C (approximately 80%) compared to HSP10-p.wt (approximately 95%). Altogether, these results suggest that the Leu73Phe mutation negatively affects the conformational stability of the HSP10 protein and impairs its spontaneous refolding.

Decreased conformational stability should render the HSP10-p.Leu73Phe protein more vulnerable to proteolytic attack. To test this we performed a limited proteolysis experiment of the HSP10-p.Leu73Phe protein at physiological temperature and pH. Purified wild type and mutant HSP10 were incubated at 37°C and pH 7.8 with trypsin for different time periods. Both the wild type and the mutant HSP10 proteins were trimmed to a shorter core fragment (HSP10*) shortly after addition of trypsin (Figure 2D). The core fragment band of HSP10-p.wt was largely stable to further attack by trypsin for up to 2 h whereas the HSP10-p.Leu73Phe core fragment band was continuously degraded resulting in strongly reduced levels already after 1 h and absence of the band after 2 h incubation (Figures 2D,E). This suggests that the core fragment is much more susceptible to proteolytic degradation when it carries a phenylalanine at the mutation site than when it carries the wild type leucine.

To elucidate which part of HSP10 was lacking in the trimmed core fragments, we performed mass spectrometric analysis of the excised full-length and core HSP10 bands (Supplementary Figure S1). Both the full-length and the core bands from wild type and mutant HSP10 showed robust MS signals for the peptides comprising amino acids 41–54 and 71–80 (peptide containing the mutation site). However, the peptide comprising amino acids 9–15 (FLPLFDR) was clearly present in the full-length band yet strongly decreased in the core bands, suggesting that low amounts of trypsin trim off N-terminal parts in both the wild type and the mutant HSP10 protein.

To rule out the possibility that the mutation impairs import of mutant HSP10 protein, we compared import of the *in vitro* labeled HSP10 wild type and mutant proteins into isolated mouse liver mitochondria. Aliquots were taken at the time points indicated and imported proteins were analyzed by SDS PAGE and phosphorimaging after reisolation of the mitochondria. Figure 2F shows the band pattern from a representative experiment. To our surprise the HSP10-p.Leu73Phe protein was clearly imported more efficiently than the wild type HSP10 protein. Adding the HSP10 proteins to the import mixture in unfolded conformation after precipitation and resuspension in denaturant resulted qualitatively in the same picture (data not shown).

### The effects of the HSP10 mutation in patient fibroblasts

**Figure 3:**
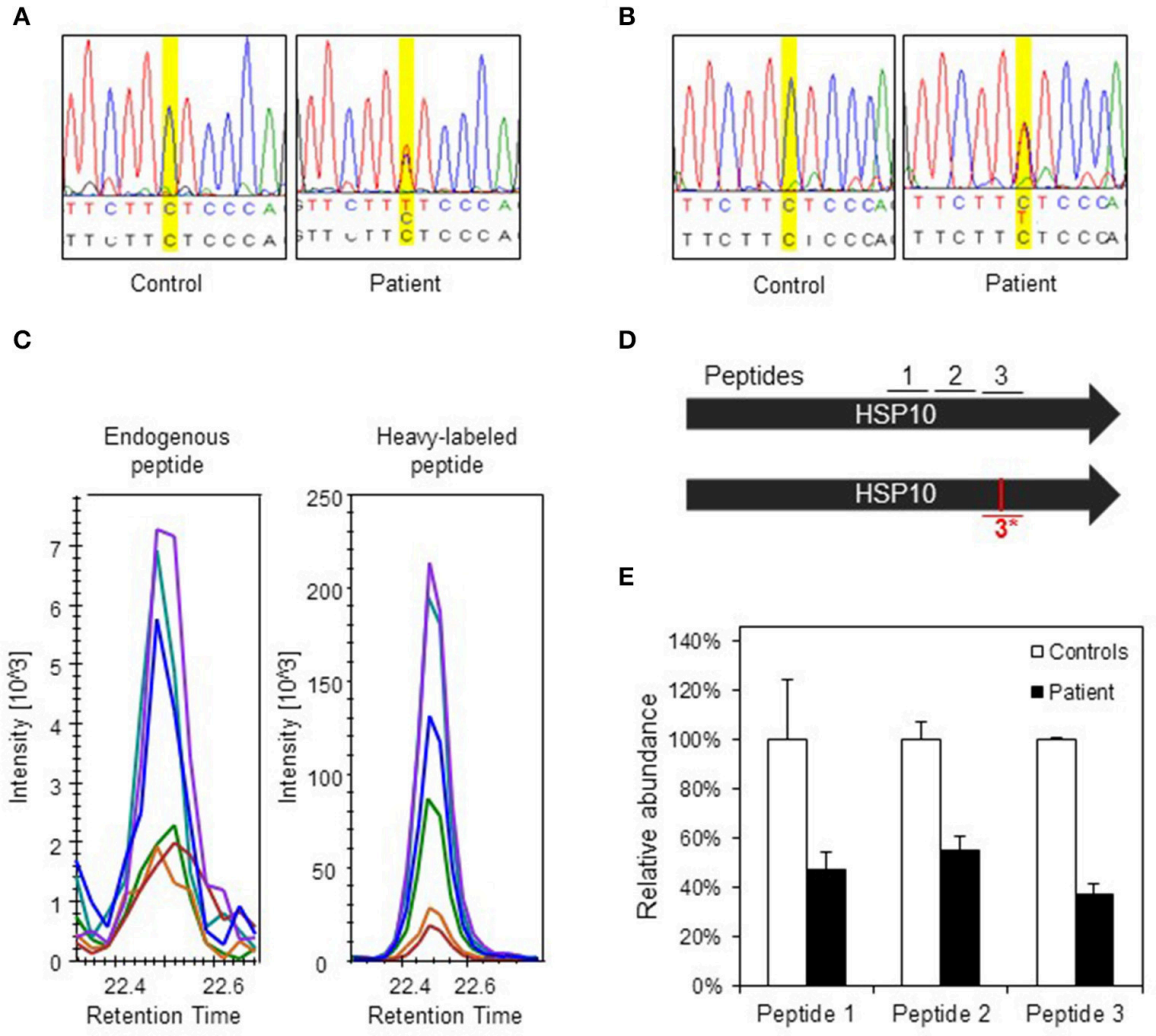
**Analysis of DNA, RNA, and protein in patient fibroblasts. (A)** Confirmation of c.217 C > T mutation in patient fibroblasts. A fragment spanning the mutation site was amplified by PCR and analyzed by Sanger sequencing. Yellow highlighting indicates position c.217 and bases are indicated below: top line depicts bases found by sequencing, lower line shows reference sequence. **(B)** Sequencing of transcripts. Total RNA was isolated from control and patient fibroblast and used for cDNA synthesis followed by PCR amplification of a fragment comprising the mutation site. PCR fragments were analyzed by Sanger sequencing. **(C)** Detection of the mutant peptide in patient fibroblasts by SRM-MS. The fragmentation patterns for the analyzed peptide fragments for the mutant peptide (#3^*^) detected in fibroblasts from the patient (left panel) and the heavy-labeled peptide that was spiked into the sample (right panel) are shown. The graphs show the peaks for the different fragment ions in corresponding colors. **(D)** Peptides used for SRM analysis. Bars show the two HSP10 alleles of the patient with position of the peptides and the mutation site. Peptides #1 and #2 are common to wild type and p.Leu73Phe HSP10 and the peptides spanning the mutation site (#3 wild type and #3^*^, mutant) are depicted. **(E)** Quantification of HSP10 in patient and control fibroblasts. The two peptides common to both wild type and p.Leu73Phe HSP10 (#1 and #2) and the peptide with wild type sequence spanning the mutation site (#3) were quantitated by SRM analysis as described in Materials and Methods. The mean of the quantitated amounts of the three peptides measured in three different control fibroblast cultures were set to 100% and the mean amounts measured in three patient cell cultures grown in parallel were expressed as percentage of these. Error bars denote standard deviation of the mean.

To evaluate the consequences of the conformational destabilization of HSP10 by the p.Leu73Phe mutation *in vivo* we turned to a cellular model system. A fibroblast culture was established from a skin biopsy of the patient. We first confirmed heterozygosity of the *HSPE1:* c.217C>T mutation by Sanger sequencing of genomic DNA isolated from the fibroblasts (Figure 3A). To elucidate whether the c.217C>T mutation affects transcript levels we isolated RNA from the fibroblast culture. Sequencing of the PCR product amplified from the reverse transcribed RNA (Figure 3B) showed a very similar tracing pattern around the mutation site as sequencing of the PCR product amplified from genomic DNA from the patient (Figure 3A). The peaks for both the C and the T at position 217 had comparable heights in Sanger sequencing of both genomic DNA and cDNA suggesting that roughly half of the *HSPE1* transcripts in the patient fibroblasts carried the mutation. The primers used for amplification of the cDNA were localized in exons 2 and 4, respectively, surrounding the mutation site in exon 3. The sequence reads of the junctions between exons 2 and 3 and between exons 3 and 4 were as expected indicating that splicing was unaffected by the mutation. qRT-PCR analysis of HSP10 and HSP60 transcript levels showed no significant differences between patient and control fibroblasts (Supplementary Figure S1). Taken together, this is consistent with the notion that the *HSPE1:*c.217C>T mutation does not significantly affect transcript levels or splicing.

To detect and quantitate the steady state levels of the HSP10-p.Leu73Phe protein in fibroblasts of the patient we applied a targeted mass spectrometric approach known as selected reaction monitoring (SRM) using heavy-labeled synthetic peptides as internal standards. Very small amounts of mutant HSP10-p.Leu73Phe protein were present in fibroblasts from the patient. Identification of this peptide was based on its fragmentation pattern that was the same as that of the heavy labeled synthetic peptide that had been spiked into the sample (Figure 3C). No corresponding peptide with such a pattern was detectable in control fibroblasts (data not shown). This indicated that small amounts of mutant HSP10-p.Leu73Phe protein were present in fibroblasts from the patient.

We then quantitated the total amounts of wild type and mutant HSP10 protein in patient and control fibroblasts using two peptides present in both the wild type and the HSP10-p.Leu73Phe protein (peptides 1 and 2 in Figure 3D). SRM analysis showed that the amounts of the HSP10 peptides 1 and 2 in the patient fibroblasts were decreased to approximately 50% as compared to the controls (Figure 3E). Measurement of the wild type peptide spanning the mutation site (peptide 3) showed only slightly lower relative amounts than peptides 1 and 2 (approximately 40% compared to approximately 50% for peptides 1 and 2) in the patient fibroblasts compared to the control fibroblasts. Taken together, our data strongly suggests that the majority of the HSP10 protein present in patient fibroblasts represents wild type HSP10 protein expressed from the wild type allele.

**Figure 4:**
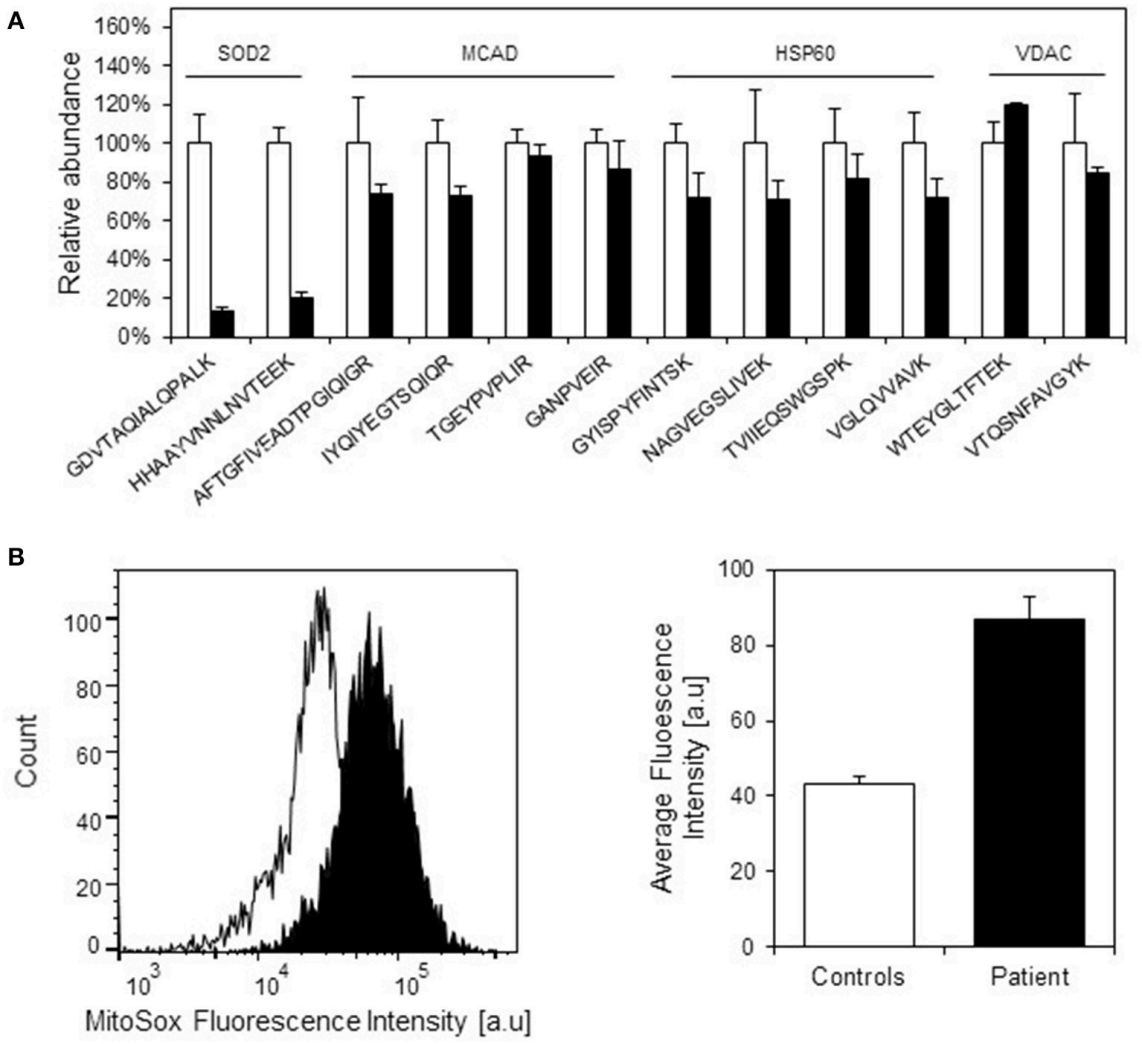
**Protein levels of selected mitochondrial proteins in patient and control fibroblasts. (A)** Levels of peptides for HSP60, MCAD, VDAC, and SOD2 were measured by SRM-MS. The quantitated amounts of all peptides measured in three different control fibroblast (open bars) were set to 100% and the amounts measured in three parallel grown patient fibroblast cultures (filled bars) were expressed as percent fraction of these. Error bars denote standard deviation of the mean. T-testing showed that the levels of the SOD2 peptides were significantly decreased (*p* < 0.05) in patient fibroblasts, while the peptides for MCAD, HSP60, and VDAC were not significantly different in patient compared to control fibroblasts. **(B)** Mitochondrial superoxide levels. Superoxide levels were determined using the fluorescent superoxide reporter MitoSOX. The left graph shows the histogram from one analysis; open peak: control, closed peak: patient. The right graph shows average mean fluorescence intensities measured from three independent cultivations and analyses of control fibroblasts and parallel cultivations of the patient fibroblast. Error bars represent SEM for the three respective replicates.

We have previously observed decreased SOD2 protein levels due to misfolding and degradation of SOD2 protein followed by increased oxidative protein damage (protein carbonylation) in a mouse model for hereditary spastic paraplegia due to HSP60 haplosufficiency (Magnoni et al., 2013). As our present investigations had shown half levels of HSP10 protein in fibroblasts from our patient, we speculated that this might also affect SOD2 protein amounts and superoxide levels. Again using SRM mass spectrometry with heavy labeled peptides as standards, we quantified SOD2 in cell lysates from patient and control fibroblasts. In parallel we quantified the complex partner HSP60, the fatty acid oxidation enzyme medium-chain acyl-CoA dehydrogenase (MCAD) that previously had been shown to interact with the HSP60/HSP10 complex (Saijo et al., 1994; Saijo and Tanaka, 1995) and the mitochondrial outer membrane protein VDAC, which was not expected to interact with the HSP60/HSP10 complex. We found comparable protein levels for HSP60, MCAD, and VDAC in patient and control fibroblasts (Figure 4A). However, the protein level of SOD2 was reduced to approximately 20% in the patient fibroblasts compared to controls. RT-qPCR analysis showed that SOD2 mRNA transcript levels, like those for the HSP60 and MCAD mRNAs, were similar in the fibroblasts from patient and controls (Supplementary Figure S2). Hence, decreased SOD2 protein levels were not due to decreased SOD2 transcript levels.

Decreased SOD2 protein levels would be expected to result in increased superoxide levels in the mitochondrial matrix. Indeed, when measuring mitochondrial superoxide with the fluorescent probe MitoSOX we observed an approximately 2-fold increase in average MitoSOX fluorescence intensity in the patient fibroblasts compared to control fibroblasts (Figure 4B). In an attempt to identify further functions that may be affected we also evaluated a number of cellular and mitochondria-related phenotypes of the patient fibroblasts. Fluorescence microscopic analysis of mitochondria labeled with Mitotracker green revealed no morphological differences between patient and control fibroblasts (Supplementary Figure S3). Furthermore, no significant differences between patient and control fibroblasts were observed in assays for cellular viability (Supplementary Figure S4A), overall oxidation state measured as the level of reduced thiols (Supplementary Figure S4B), and mitochondrial membrane potential (Supplementary Figure S4C). We finally analyzed OCR as a measure for mitochondrial respiratory chain activity using a Seahorse extracellular flux analyzer. No significant differences in basal OCR, reserve capacity, and ATP-linked respiration (Supplementary Figure S4D) as well as basal extracellular acidification rate (data not shown) between patient and control fibroblasts were observed. This suggests that HSP10 deficiency did not have a major effect on mitochondrial respiratory chain activity and balance between mitochondrial respiration and anaerobic glycolysis.

## Discussion

Exome sequencing of the patient with infantile spasms and developmental delay described here detected heterozygosity for a *de novo* mutation in the *HSPE1* gene and chromosomal microarray analysis detected a *de novo* deletion affecting the *TANC2* gene. Expression levels of the TANC gene family genes *TANC1* and *TANC2* have been indicated to regulate the density of dendritic spines and excitatory synapses (Han et al., 2010) and diagnostic exome sequencing identified a *de novo* TANC1 missense variation in one patient with severe intellectual disability (de Ligt et al., 2012). Both the HSP10 missense mutation and the loss of one *TANC2* allele may potentially be disease-associated. Because the *HSPE1* gene encoding the small subunit of the mitochondrial HSP60/HSP10 chaperonin complex is essential for cell function and mutations in its complex partner HSP60 have been associated with neurological diseases, we have in the present study focused on investigating the effects of the HSP10-p.Leu73Phe mutation.

The odds for accidentally finding a *de novo* mutation in a specific gene are small. Data from exome sequencing predict on average one *de novo* missense mutation per exome per generation (Veltman and Brunner, 2012; Alkuraya, 2016). Given this number, the length of the HSP10 coding region with 306 bases, the total human exome length with approximately 3.10^7^ bases, and the world population at currently approximately 7.5 × 10^6^ people, one can statistically expect 27 currently living individuals carrying a *de novo* mutation in the *HSPE1* exome. A considerable fraction of these would be synonymous, so only a handful of individuals will have missense or premature stop codon variations in the *HSPE1* gene.

Inspection of the mutation site in the structure of the human HSP60/HSP10 complex showed that leucine-73 of HSP10 is not localized in the domain that interacts with HSP60 subunits in the complex. Rather, it is situated in the central part of the HSP10 structure, tightly packed by residues within the same HSP10 subunit. The phenylalanine that replaces leucine in the mutant protein can apparently be accommodated in the structure, but, because it is more bulky, it requires rearrangements. Such rearrangements of the domain are expected to impair folding and stability. Our *in vitro* analysis of recombinant HSP10-p.Leu73Phe protein showed that although this protein could be recombinantly expressed and purified in a fully functional form, its thermal stability was indeed profoundly decreased and its spontaneous refolding after denaturation was impaired. Although, the decreased melting temperature of the HSP10-p.Leu73Phe protein determined under *in vitro* conditions was well above 37°C, we show that the mutant HSP10 protein displays increased susceptibility to proteolytic attack at 37°C and physiological pH.

Our observation that posttranslational import of the HSP10-p.Leu73Phe protein into isolated mitochondria was more efficient than import of the wild type HSP10 protein was surprising. However, it has been shown for some other proteins that mutations destabilizing the conformational structure of precursor proteins in the cytosol enhances their post-translational import into mitochondria (Vestweber and Schatz, 1988). Protein import through the mitochondrial outer and inner membrane as well as degradation by the ATP dependent proteases Lon and ClpXP in the mitochondrial matrix require previous unfolding of the transported/degraded polypeptide (Lee et al., 2001; Prakash and Matouschek, 2004; Maurizi and Stan, 2013). Thus, what at first sight appears to be counterintuitive, likely reflects that the decreased conformational stability renders the mutant HSP10 protein both more prone to be transported through the mitochondrial import system and more prone to degradation by mitochondrial proteases. In line with this, the steady state levels of the HSP10-p.Leu73Phe protein in the patient fibroblasts were much lower than those of the HSP10-p.wt protein. Because transcript levels of the mutant allele appeared unchanged and—given its position in the transcript—because an effect of the mutation on translation is unlikely, this suggested that the decreased level of the HSP10-p.Leu73Phe protein is due to higher susceptibility to proteolytic degradation.

Given the measured decreased Hsp10 to HSP60 protein ratio, we wondered what effects this could potentially have? The genes encoding HSP60 and HSP10, respectively, of eukaryotes from *C. elegans* to humans are organized in a head-to-head arrangement with a common bidirectional promoter (Ryan et al., 1997; Bross et al., 2013). Furthermore, in bacteria the corresponding genes encoding the large and small chaperonin subunits are typically organized in an operon under control of a common promoter (Lund, 2009). These architectures may provide transcription of both chaperonin genes at a fixed ratio suggesting that this ratio is crucial for correct function of chaperonin complexes. It has indeed been shown that the kinetics of *in vitro* refolding of malate dehydrogenase by the HSP60/HSP10 complex depended on the HSP10 to HSP60 ratio (Levy-Rimler et al., 2001). Maximum refolding rate was obtained at approximately 2:1 HSP10: HSP60 molar ratio and clearly lower refolding was observed at a ratio of to 1:1. Levy-Rimler et al. also presented evidence that HSP10 stabilizes the double-ring conformation of human HSP60. Thus, the observed almost 2-fold decrease of the HSP10/HSP60 ratio would be expected to compromise the function and capacity of the HSP60/HSP10 complex to assist folding and result in significant impairment of folding of those proteins that are most dependent on folding assistance by the HSP60/HSP10 complex.

We have previously shown that folding of SOD2, the superoxide dismutase in the mitochondrial matrix, was dramatically compromised in mice that express half levels of HSP60 due to heterozygosity for a knock-out allele of the murine *Hspd1* gene (Magnoni et al., 2014). Furthermore, we could demonstrate physical interaction of SOD2 with the HSP60/HSP10 complex. As expected, our analyses of the patient fibroblasts studied here showed that SOD2 protein levels were significantly decreased. Decreased SOD2 protein levels were not caused by decreased *SOD2* transcript levels as shown by qRT-PCR analysis. This is consistent with the notion that SOD2 is one of the proteins in the mitochondrial matrix, which particularly depend on the HSP60/HSP10 complex to acquire its native state. The native state of the SOD2 enzyme is a homotetramer with a manganese atom in the active site of all four subunits (Borgstahl et al., 1992). Decreasing either component of the HSP60/HSP10 complex appears to impair SOD2 protein folding and entail degradation of SOD2 folding intermediates. Recombinant expression experiments in *E. coli* have shown that yield, metal content and activity of human SOD2 were increased when the bacterial HSP60/HSP10 homolog GroEL/GroES was co-overexpressed (Hunter and Hunter, 2013). Assistance by the HSP60/HSP10 chaperonin complex for folding and concomitant metal incorporation may be a particularly crucial step that it is impaired when the capacity of HSP10/HSP60 complex is limiting.

No monogenic human disease caused by mutations in the *SOD2* gene has been described yet. A series of knockout mouse models for the *Sod2* gene have been investigated mostly in context with research of the mitochondrial free radical theory of aging (Barja, 2013). Knock-out of both *Sod2* alleles leads to lethality within 18 days of birth in different strain backgrounds (reviewed in Marecki et al., 2014). Mice with only one *Sod2*-knock-out allele that express half levels of the SOD2 protein had normal life-span and no overt phenotype, but displayed increased oxidative damage as well as increased apoptosis tendency (Strassburger et al., 2005; Wenzel et al., 2008). The SOD2 levels we measured in the patient fibroblasts were less than 50% of controls suggesting more severe consequences than those modeled in the heterozygous SOD2 knockout mice. Damage caused by high levels of reactive oxygen species is especially critical for neuronal tissue that only to very limited extent can be regenerated. A consequence of decreased SOD2 activity would be increased superoxide levels in the mitochondrial matrix and we indeed measured a significant increase in matrix superoxide using the mitochondrial matrix fluorescence reporter MitoSOX. This may advocate that increased superoxide levels represent one of the disease-associated molecular phenotypes in the current case.

The question remains whether increased superoxide levels are the only major effect of the HSP10 mutation in the patient cells investigated in our study. The observation of intermittently increased levels of various disease marker metabolites related to mitochondrial enzyme deficiencies in the patient suggests that the SOD2 deficiency is probably only one of several consequences of the skewed HSP10/HSP60 ratio. Our further analysis of the patient fibroblasts for general mitochondrial factors like mitochondrial membrane potential, cellular redox status and mitochondrial respiratory chain activity showed no significant differences compared to controls suggesting that potential additional effects on these parameters are subtle or not manifesting in fibroblasts.

We also analyzed whether the levels of medium-chain acyl-CoA dehydrogenase (MCAD) were affected in the patient cells. MCAD is an enzyme involved in mitochondrial fatty acid oxidation that has previously been shown to interact with the HSP60/HSP10 complex (Yokota et al., 1992; Saijo et al., 1994). Yet, our investigations showed similar MCAD protein levels in the patient and control fibroblasts, suggesting that folding of this interactor is less affected by the decreased HSP10 levels. The interactors of the homologous bacterial GroEL/GroES complex have been characterized and distinguished into classes depending on the length of the time period they interact (Ewalt et al., 1997). Among approximately 250 interactors a core set of about 85 proteins was shown to be highly dependent on folding assistance by the chaperonin complex (Kerner et al., 2005). Such an inventory of interactors and validation of their dependence on the function of the HSP60/HSP10 complex would be helpful for pinpointing further candidates, but is still lacking for the mammalian HSP60/HSP10 complex.

In conclusion, as we did not investigate potential effects of the deletion of parts of the *TANC2* gene, we can currently not exclude that it is disease associated. However, based on the results from a battery of *in vitro* and *ex vivo* studies, we make the case that heterozygosity for the HSP10-p.Leu76Phe *de novo* missense mutation potentially could be responsible for the neurological and developmental disorder of the patient reported here.

## Author contributions

AB, PB, AA, PF, KH, JP, and TC designed the experimental setup. KH, LB, have performed the clinical analysis of the patient. JD, HL, ND performed the clinical exome sequencing, chromosome microarray analysis and interpretation of these data. AB, SN, XL, PF, DP, RB, JP, and PB performed the experimental work. All authors were involved in writing the first draft and finalizing the manuscript.

### Conflict of interest statement

The authors declare that the research was conducted in the absence of any commercial or financial relationships that could be construed as a potential conflict of interest.

## Supplementary material

Supplementary Figure S1 **Mass spectrometric analysis of HSP10 full-length and core bands. (A)** Full-length and core bands from a limited proteolysis experiment like the one shown in Figure 2D were excised and prepared for mass spectrometry (LC-MS/MS). Elution profiles for detected peptides are shown in **(A)**. The arrows point to peptides that are highlighted in the corresponding colors in **(B)**. Green arrow: amino acids 41–54 (VLQATVVAVGSGSK), magenta arrows: amino acids 71–80 (VL-x-PEYGGTK with Leu (wt) or Phe (mutant), respectively, at the “x”-position), blue arrow: amino acids 9–15 (FLPLFDR). **(B)** HSP10 subunit R in schematic representation. Position of lysine and arginine residues are highlighted in yellow, the mutated leucine-73 residue is shown as sticks in red and the amino (N) and carboxy-terminal (C) positions are depicted. The representation was made with Discovery Studio Visualizer v.4.5 (Biovia) using PDB coordinates for the human HSP60/HSP10 complex (4PJ1).

Supplementary Figure S2 **RT-qPCR quantitation of transcript levels**. RNA purified from patient and three individual control fibroblasts was reverse transcribed into cDNA and subjected to quantitative analysis with qPCR TaqMan assays. Error bars represent standard error of mean for biological triplicates. Filled bars: Patient, open bars: mean from three pooled controls. *P*-values were obtained with student's *t*-test and showed no significant differences.

Supplementary Figure S3 **Mitochondrial morphology**. Fibroblasts from the patient and a healthy control individual were seeded at 50% confluence in 6-well plates 24 h before the analysis. The fibroblasts were stained with 100 nM Mitotracker Green for 30 min at 37°C. Pictures were obtained with the EVOS FLoid Cell Imaging Station as described in materials and methods. Two representative pictures each of the control (left panel) and the patient (right panel) fibroblasts are shown.

Supplementary Figure S4 **Cellular viability, thiol redox status, mitochondrial membrane potential, and mitochondrial oxygen consumption profiling. (A)** Cellular viability measured as the percentage of viable cells. **(B)** Cellular thiol redox status measured as intensity of the Vita-Bright 48 (V.B-48) fluorescent dye. V.B-48 becomes fluorescent when it reacts with reduced thiols which are used to quench molecules that are damaging to the cell, such as free radicals, and are used as a measurement for general cellular oxidative/reduced status. **(C)** The mitochondrial membrane potential measured as the red/green ratio of the fluorescent dye JC-1. An assay control (control cells treated with the uncoupler CCCP) is also shown. **(D)** Mitochondrial oxygen consumption rate profiling. Oxygen consumption rate (OCR) of patient and control fibroblasts in dependence of addition of modulators [1: oligomycin (inhibitor of complex V); 2: FCCP (uncoupler); 3: antimycin A and rotenone (inhibitors of complex I and III, respectively)] was measured using the Mito Stress test-kit and the Seahorse XF^*e*^96 extracellular flux analyzer. Samples from 3 different control fibroblasts and 3 independently grown cultures of the patient fibroblasts were analyzed. OCR was normalized to total protein amount quantified by the Bradford assay.

Supplementary Table S1 **Oligonucleotide primer sequences**.

Supplementary Table S2 **Genes investigated in exome sequencing**.

## Abbreviations

CSF: cerebrospinal fluid
PQC: protein quality control
SRM: selected reaction monitoring.

## Appendix

### Extended clinical patient information

The patient presented at 3 months of age with spastic movements. An EEG diagnosed infantile spasms. A brain MRI did not show any abnormalities. At the age of 3, he was diagnosed with myoclonic and tonic epilepsy. He came to the attention of medical genetics at 7 months of age. Developmental milestones were globally delayed. He rolled over at 7 months, sat at 16 months, and walked at 3½years. At 17 months he said only a single word. At the age of 4, he is still largely nonverbal. He was found to have intermittent exotropia and cortical visual impairment on ophthalmologic examination. Mild hepatomegaly and slightly course features were noted by neurology, prompting the lysosomal screening. These were not appreciated by genetics. Aside from mild macrocephaly and a thin upper lip, the patient did not demonstrate dysmorphic features on examination by genetics.
