# Supplementary material for: Effects of a Mutation in the HSPE1 Gene Encoding the Mitochondrial Co-chaperonin HSP10 and Its Potential Association with a Neurological and Developmental Disorder
Source: Front Mol Biosci. 2016 Oct 7;3:65. doi: 10.3389/fmolb.2016.00065 (PMC5053987; doi:10.3389/fmolb.2016.00065)
Supplement: Supplementary Figure S1 — Mass spectrometric analysis of HSP10 full-length and core bands. (A) Full-length and core bands from a limited proteolysis experiment like the one shown in Figure 2D were excised and prepared for mass spectrometry (LC-MS/MS). Elution profiles for detected peptides are shown in (A). The arrows point to peptides that are highlighted in the corresponding colors in (B). Green arrow: amino acids 41–54 (VLQATVVAVGSGSK), magenta arrows: amino acids 71–80 (VL-x-PEYGGTK with Leu (wt) or Phe (mutant), respectively, at the “x”-position), blue arrow: amino acids 9–15 (FLPLFDR). (B) HSP10 subunit R in schematic representation. Position of lysine and arginine residues are highlighted in yellow, the mutated leucine-73 residue is shown as sticks in red and the amino (N) and carboxy-terminal (C) positions are depicted. The representation was made with Discovery Studio Visualizer v.4.5 (Biovia) using PDB coordinates for the human HSP60/HSP10 complex (4PJ1). [file Presentation1.PPTX]

## Slide 1
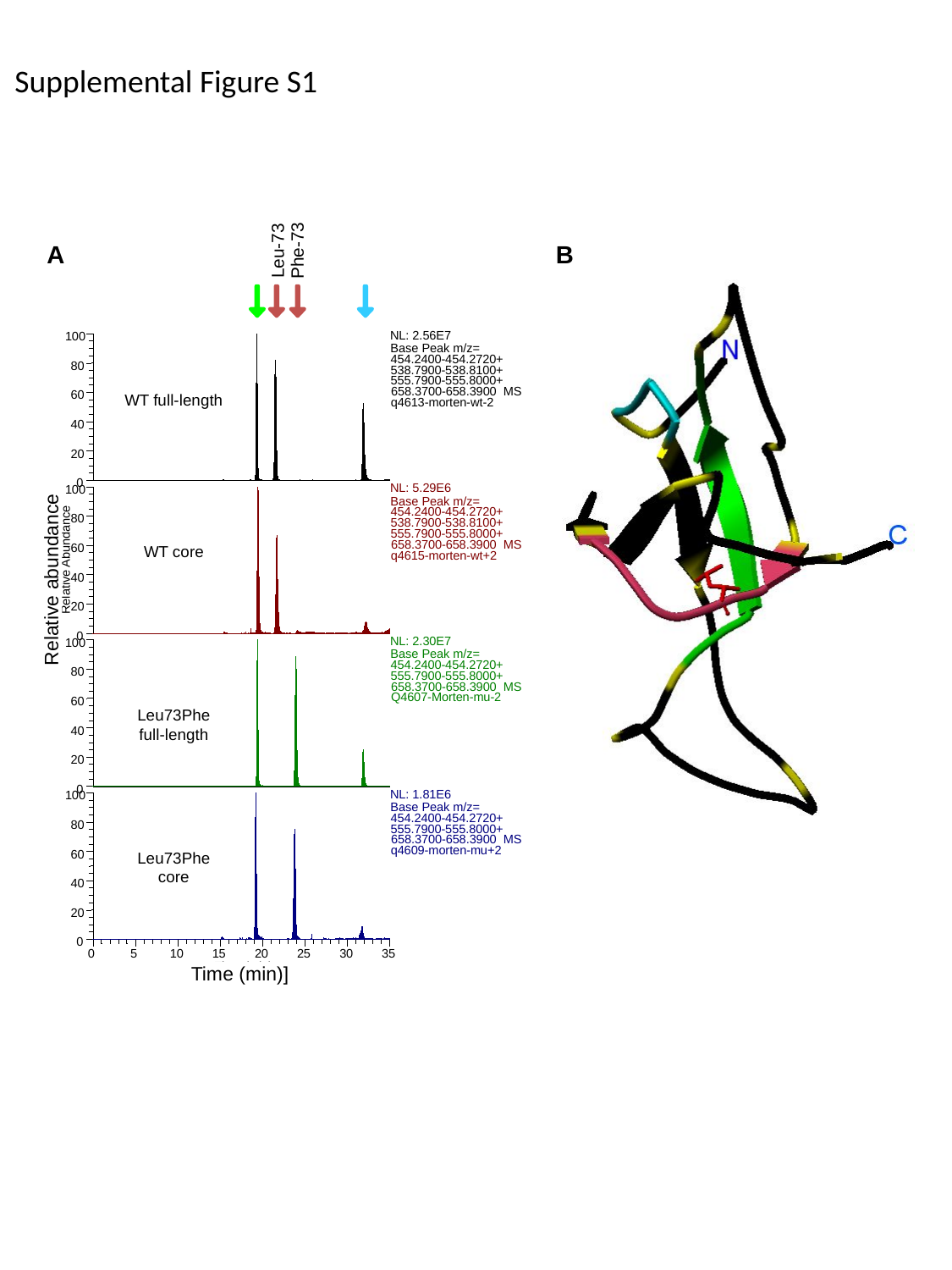

Supplemental Figure S1
A
B
Phe-73
Leu-73
NL: 2.56E7
100
Base Peak m/z=
454.2400-454.2720+
80
538.7900-538.8100+
555.7900-555.8000+
658.3700-658.3900 MS
60
q4613-morten-wt-2
40
20
0
NL: 5.29E6
100
Base Peak m/z=
454.2400-454.2720+
80
538.7900-538.8100+
555.7900-555.8000+
658.3700-658.3900 MS
60
q4615-morten-wt+2
Relative Abundance
40
20
0
NL: 2.30E7
100
Base Peak m/z=
454.2400-454.2720+
80
555.7900-555.8000+
658.3700-658.3900 MS
Q4607-Morten-mu-2
60
40
20
0
NL: 1.81E6
100
Base Peak m/z=
454.2400-454.2720+
80
555.7900-555.8000+
658.3700-658.3900 MS
q4609-morten-mu+2
60
40
20
0
0
5
10
15
20
25
30
35
Time (min)
WT full-length
WT core
Relative abundance
Leu73Phe
full-length
Leu73Phe
core
Time (min)]

## Slide 2
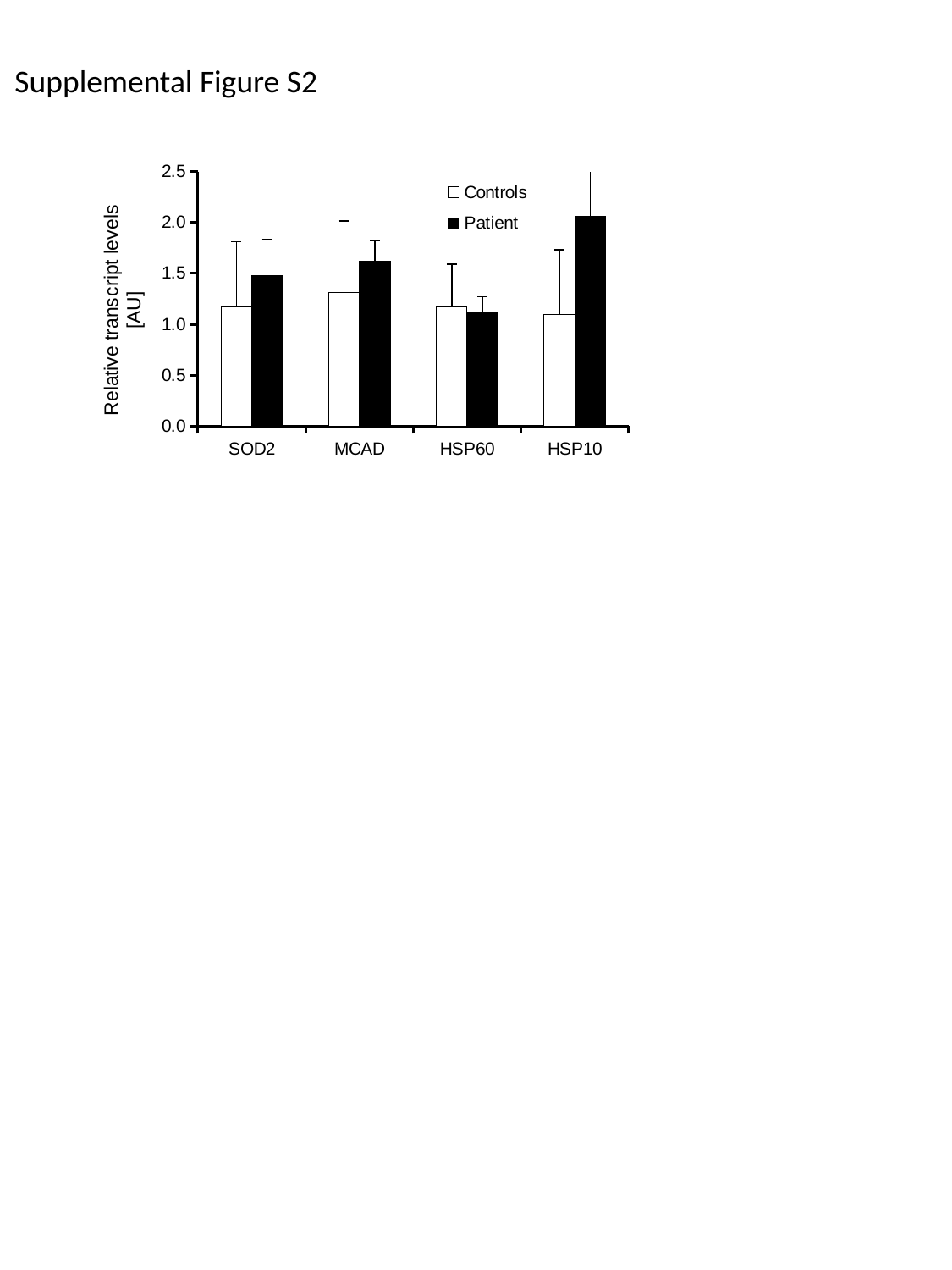

Supplemental Figure S2
### Chart
| Category | Controls | Patient |
|---|---|---|
| SOD2 | 1.1753289500872295 | 1.4766212304433186 |
| MCAD | 1.3084806005160015 | 1.6162593364715576 |
| HSP60 | 1.1732866168022156 | 1.1120009024937947 |
| HSP10 | 1.0926427841186523 | 2.0583566427230835 |

## Slide 3
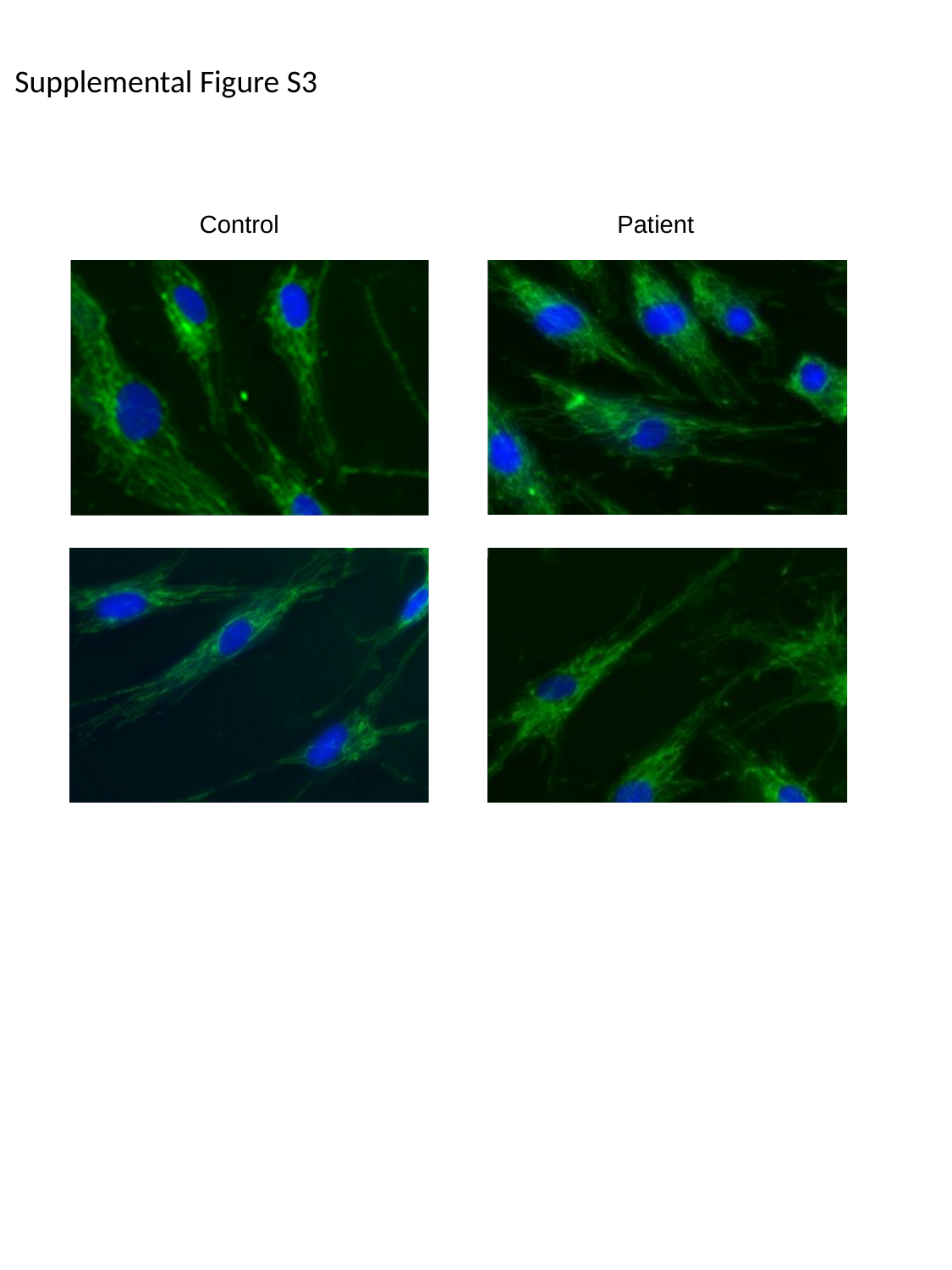

Supplemental Figure S3
Control
Patient

## Slide 4
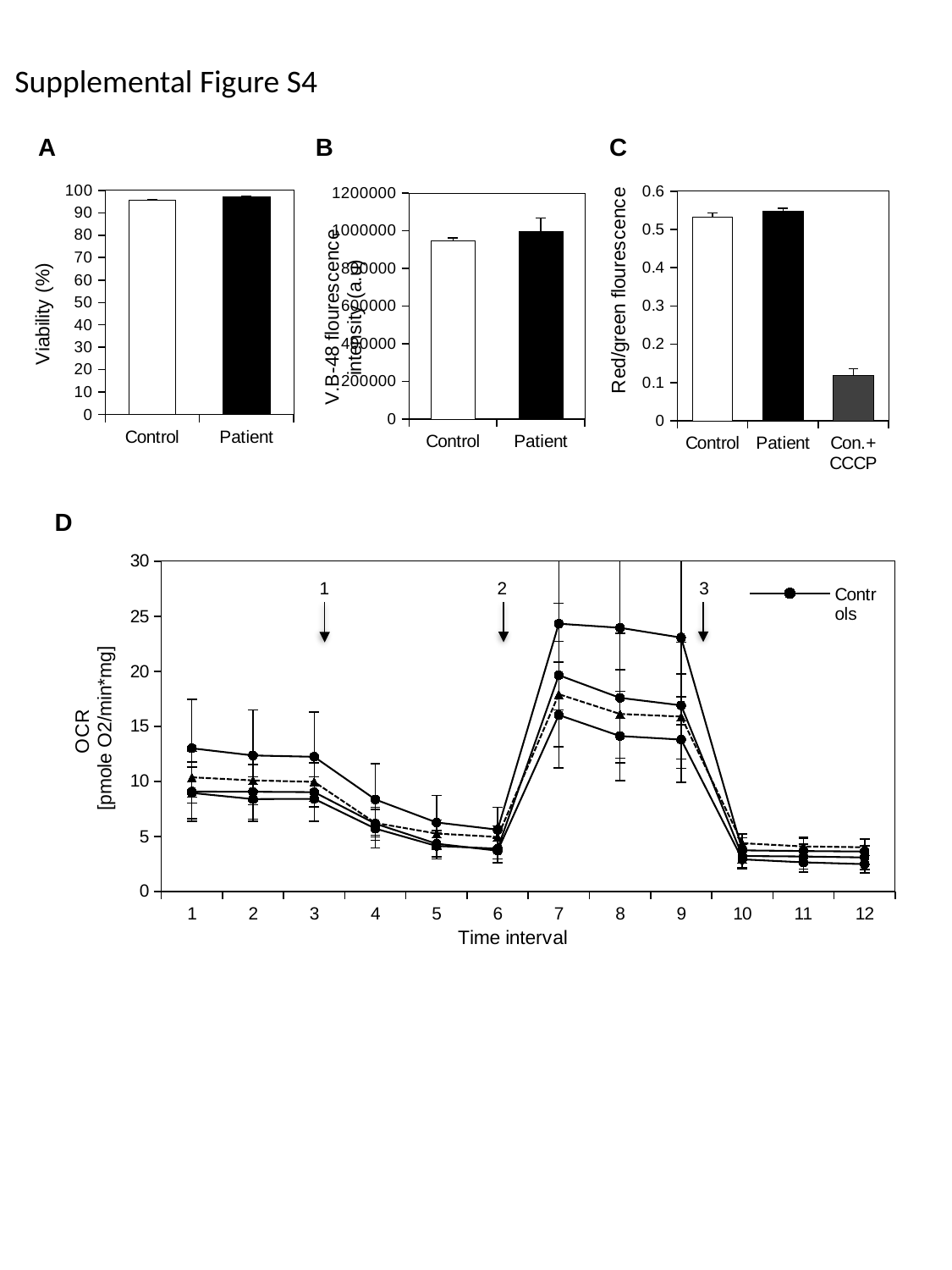

Supplemental Figure S4
A
B
C
### Chart
| Category | |
|---|---|
| Control | 0.5331665628287118 |
| Patient | 0.5486432733592831 |
| Con.+ CCCP | 0.11770988646438214 |
### Chart
| Category | |
|---|---|
| Control | 95.56111111111112 |
| Patient | 97.05555555555556 |
### Chart
| Category | |
|---|---|
| Control | 947019.9222222221 |
| Patient | 996679.2777777779 |D
### Chart
| Category | NHDF-01 | NHDF-06 | | |
|---|---|---|---|---|1
2
3
