# Supplementary material for: Effects of a Mutation in the HSPE1 Gene Encoding the Mitochondrial Co-chaperonin HSP10 and Its Potential Association with a Neurological and Developmental Disorder
Source: Front Mol Biosci. 2016 Oct 7;3:65. doi: 10.3389/fmolb.2016.00065 (PMC5053987; doi:10.3389/fmolb.2016.00065)
Supplement: Supplementary Table S1 — Oligonucleotide primer sequences. [file DataSheet1.docx]

**Supplementary tables:**

**Supplementary Table S1: Oligonucleotide primer sequences**

| **Primer** | **sequence** |
| --- | --- |
| Intron-2 sense  Exon-4 UTR antisense  Exon-2 sense | 5’-CCATGTTTCATTAGTTGTAGTG–3’  5’-AGATTTCAGAACTTCAGTGGA-3’  5’-TTGCAAGCAACAGTAGTCGC-3’ |
| Exon-4 antisense  m HSP10L73F_F  m HSP10L73F_R | 5’-TTCCAAGAATGTCACCATCTCT-3’  5’-GTTGGAGATAAAGTTCTTTTCCCAGAATATGGAGGC-3’  5’-GCCTCCATATTCTGGGAAAAGAACTTTATCTCCAAC-3’ |

| **Supplementary table S2: Genes investigated in exome sequencing** |
| --- |
| ABAT, ABCC8, ABCD1, ABHD12, A CADS, A C02, ACOX1, ACP2, ACP5, ACSF3, ACTB, ACVR1, ACY1, ADARB1, ADCK3, ADCYAP1R1, ADK, ADSL, AFF2, AFG3L2, AFP, AGL, AGPS, AGTR2, A/MP1, AKAP5, AKT3, ALDH4A1, ALDH5A1, ALDH7A1, ALDOA, ALG11, ALG6, ALG9, ALPL, ALS2, ALX3, ALX4, AMACR, AMER1, AMPH, ANG, ANK1, ANKH, AP481, AP4E1, AP4S1, APOA1, APOE, APOLD1, APP, AQP4, ARHGAP21, ARHGAP9, ARHGEF9, AR/018, ARRDC3, ARSA, ARX, ASAH1, ASPA, ASPM, ATAD1, ATF2, ATL1, ATN1, ATOX1, ATP11B, ATP1A2, ATP1A3, ATP6, ATP6VOA2, ATP7A, ATP8, ATPAF2, ATR, ATRX, AUH, AUTS2, BAK1, BCAP31, BCKDK, BCS1L, BDNF, BIN!, BMP4, BMPR1A, BRAF, BRWD3, BSCL2, BSN, C10orf2, C19orf12. C3, C3orf58, CACNA1A, CACNA1G, CACNA1H, CACN84, CACNG2, CAMTA1, CANT1, CARD8, CASK, CASR, CBL, CC202A, CCT5, CD96, CDK19, CDK5R1, CDK5RAP2. CDKL5, CDNF, COON, CENPJ, CEP164, CEP57, CFC1, CFL1, CGNL1, CHAT, CHD2, CHD7, CHD8, CHMP1A, CHRM3, CHRNA4, CHRNA7, CHRNB2, CIT, CLCN2, CLN5, CLN6, CMIP, CNR1, CNTN5, CNTN6, CNTNAP2, CNTNAP3, COG4, COG6, COG?, COL11A1, COL11A2, COL 18A1, COL1A2, COL2A1, COL3A1, COL4A1, COL4A2, COQ2, COQ6, COX15, COX3, COX681, CPA6, CPLX1, CPLX2, CPT2, CRB1, CREB1, CREBBP, CRH, CRLF1, CRYAB, CSF1R, CSTB, CTC1, CTNNB1, CTSA, CTSD, CTSF, CUL4B, CYB5R3, CYP11A1, CYP19A1, CYP7B1, CYTB, D2HGDH, DARS2, DBP, DCLK2, DCX, DDHD2, DECR1, DFNB31, DGKD, DHCR24, DHFR, DIS3, DISC1, DMD, DNAJB6, DOCK8, DOCK9, DOPEY2, DPAGT1, DPYS, DRD5, DYNC1H1, DYRK1A, EARS2, EBP, ECEL1, EFHC1, EFNB1, EHMT1, EIF2B4, EIF2B5, ELN, ELOVL4, EMX2, EOMES, EPG5, EPHA7, EPHX1, EPM2A, ERCC6, ERL/N2, ERMN, ETFDH, F2, FA2H, FAM58A. FARS2, FBN1, FBN2, FBX041, FBX07, FGD1, FGF2, FGF22, FGF7, FGF8, FGFR1, FGFR2, FGFR3, FGFRL1, FH, FKTN, FLNA, FMR1, FNDC1, FOLR1, FOS, FOXC1, FOXG1, FOXP1, FOXP2, FOXRED1, FTL, FXN, GABBR1, GABRA4, GABRB3, GABRD, GABRG2, GAD1, GAD2, GALC, GALE, GAMT, GAN, GAS1, GATA6, GATAD2B, GBA, GCDH, GCH1, GCK, GDI1, GFAP, GFM1, GHR, GJC2, GK, GLB1, GLDC, GL/2, GL/3, GLRB, GL YCTK, GM2A, GNB5, GNG3, GNPA T, GNS, GOSR2, GPC4, GPR61, GPR84, GPR85, GPR98, GPRC5B, GPRIN1, GPRIN2, GPSM2, GRIA1, GRIA2, GRIA3, GRIA4, GRIK1, GRIK2, GRIN1, GRIN2A, GRIN2B, GRM1, GRN, GTF21, HAX1, HBB, HCN2, HDAC4, HDAC8, HECW1, HEPACAM, HESX1, HEXA, HIBCH, HLF, HMG20B, HMGA2, HMGB1, HNRNPU, HPSE, HRAS, HSD17B10, HSPD1, HTR2C, HUWE1, HYDIN2, ICAM5, ICK, IDS, IDUA, IFT140, IGBP1, IGSF11, IGSF9B, IL10, IL1B, IL1RAPL 1, IL1RN, IL6, INTS2, INTU, ISLR2, ISPD, ITPR1, JAG1, JAM3, JRK, JRKL, JUN, KANSL1, KAT6A, KCNA1, KCNAB2, KCNH3, KCNJ10, KCNJ11, KCNJ2, KCNJ6, KCNMA1, KCNQ2, KCNQ3, KCNT1, KCT013, KCTD7, KIAA0196, KIAA0415, KIAA1109, KIF1A, KIF5A, KIF?, KLHL2, KRAS, KRIT1, L1CAM. LAMA2, LAMC3, LCOR, LETM1, LG/1, LHX4, LIAS, LILRA3, LINC00237, LRFN2, LRFN5, LRP1, LRP2, LRRTM1, LRRTM2, LRRTM3, LRRTM4, MAGI2, MAOA, MAOB, MAPT, MARCKS, MARS2, MAST1, MAU2, MBD5, MBTPS2, MCCC2, ME2, MECP2, MED12, MED17, MEF2C, MFRP, MFSD8, MIPOL1, MKS1, MLC1, MMADHC, MOCS2, MRAP, MRPS16, MRPS22, MSM01, MSX2, MTHFR, MTMR2, MTPAP, MTR, MTUS1, MYCN, MYH14, NAGLU, NBN, NBPF1, ND1, ND3, ND4, ND5, ND6, NDP, NDRG2, NDUFA1, NDUFA2, NDUFAF1, NDUFAF3, NDUFAF5, NDUFAF6, NDUFS8, NEUROD1, NEUROD2, NF1, NFI A, NFIX, NGL Y1, NHLRC1, NHS, NIP A 1, NIPBL, NKAIN2, NLGN3, NLGN4X, NLN, NMNAT1, NMNAT2, NNT, N002, NOG, NOL3, NOTCH2, NOTCH4, NPEPPS, NPHP3, NRAS, NRG3, NRXN1, NSD1, NSOHL, NSUN2, NTF4, NTRK2, NTSR2, NUBPL, OFD1, OPA1, OPHN1, OTC, OTX1, OTX2, P2RY12, PAFAH1B1, PANK2, PAX2, PAX6, PCCB, PCDH11X, PCDH11Y, PCDH19, PCMT1, POCD10, PDE4D, PDGFB, PDHA1, POHX, PDYN, PEX13, PEX16, PEX19, PEX3, PEX5, PGK1, PHIP, PHRF1, PIGA, PIGL, PIGM, PIGN, PIGV, PITX2, PLA2G6, PLCB1, PLEKHA6, PLEKHG4, PLP1, PMP22, PNKP, PNPO, POC1A, POLG, POLG2, POLR3A, POLR3B, POMGNT1, POMT1, POMT2, POU1F1, PPARD, PPT1, PPT2, PQBP1, PRICKLE1, PRICKLE2, PRNP, PROOH, PROS1, PRRT2, PSAP, PSAT1, PSEN1, PTCH1, PTEN, PTH, PTPN11, PURA, PYCR1, QDPR, QK/, RAB39B, RAD21, RA/1, RALGAPA1, RANBP2, RAPSN, RBFOX1, RDH12, REEP1, RELN, RIN2, RMND1, RNASET2, RNF135, RNF213, RNU4ATAC, ROGDI, ROR2, RPGR/P1L, RPS6KA3, RRM2B, RTN2, RTTN, RUNX1, RXFP3, RYR2, RYR3, SACS, SAMHD1, SATB2, SCARB2, SCN1A, SCN1B, SCN2A, SCN2B, SCN3A, SCN8A, SCN9A, SDHA, SDHB, SEPSECS, SERAC1, SERINC2, SERINC5, SERP/N/1, SEZ6, SGCE, SH2B1, SHANK2, SHANK3, SHC3, SHH, SHOC2, SHROOM4, S/GMAR1, S/L1, S/X3, SLC12A2, SLC12A5, SLC12A6, SLC13A1, SLC16A2, SLC17A5, SLC17A7, SLC19A3, SLC1A2, SLC1A3, SLC25A11, SLC25A12, SLC25A15, SLC25A20, SLC25A22, SLC25A33, SLC25A42, SLC25A44, SLC25A46, SLC2A1, SLC33A1, SLC39A6, SLC44A1, SLC46A1, SLC4A10, SLC4A5, SLC5A2, SLC6A1, SLC6A16, SLC6A5, SLC6A7, SLC6A8, SLC9A6, SLC9A9, SMARCA2, SMCJA, SNN, SNORD116-1, SOD1, SOS1, SOX10, SOX2, SPAST, SPG11, SPG20, SPG7, SPR, SPTAN1, SRGAP2, SRPX2, SRR, ST3GAL3, ST5, ST8S/A1, STAT!, STK11, STRA6, STS, STX1A, STXBP1, SUFU, SULF1, SV2A, SYN1, SYT14, TAAR5, TAC1, TANC1, TAOK1, TBC101, TBC1024, TBCD, TBP, TBX1, TBX2, TCF4, TDGF1, TEF, TFAP2B, TGFBR2, TG/F1, THRB, TLR4, TMEM1320, TMEM67, TNF, TNFRSF1A, TNFRSFJB, TNS3, TP/1, TPK1, TPP1, TRAPPC9, TRIM2, TRIM3, TRMT5, TRNC, TRNF, TRNH, TRNK, TRNL1, TRNN, TRNR, TRNV, TRNW, TRPM6, TSC1, TSC2, TSEN54, TSHZ1, TTR, TUBA1A, TUBA8, TUBB2B, TUBB3, TULP3, UBE2A, UBE3A, UCP2, UGT2B17, UPF3B, VAMP1, VAX1, VPS37A, WASF3, WDR35, WDR48, WDR62, WDR81, WHSC1, XIST, XPNPEP3, YEATS2, YWHAE, YWHAG, ZDHHC15, ZDHHC9, ZEB2, ZFP57, ZFYVE26, ZFYVE27, ZIC2, ZNF238, ZNF259, ZNF333, ZNF423, ZNF687, ZNF81 |
